# Case Report: Accidental firearm injury during trophy hunting and the role of paramedics in managing such cases at rural health posts in Nepal

**DOI:** 10.12688/f1000research.55659.2

**Published:** 2022-03-04

**Authors:** Alok Atreya, Samata Nepal, Ashal Timalsina, Geeta Bashyal, Lokaratna Gyawali, Jenash Acharya

**Affiliations:** 1 Lumbini Medical College, Palpa, Lumbini, 32500, Nepal; 2 District Hospital, Palpa, Lumbini, 32500, Nepal; 3 Kathmandu Medical College Teaching Hospital, Kathmandu, Bagmati, 44600, Nepal

**Keywords:** Accidental injuries; wounds, gunshot; wounds, penetrating

## Abstract

Possession of a firearm without a certified valid license is against Nepalese law. Following a decade-long civil war, Nepal government issued stringent laws not allowing public to possess firearms without a valid reason, despite bearing a license. However, there are people who possess and use firearms for hunting purposes. The present case reports an accidental death of a teenage boy who used a musket for hunting. The present case highlights the fact that despite these stringent laws, illegal possession of arms for trophy hunting is still prevalent in rural Nepal. Furthermore, this study aims to highlight the importance of paramedics in early intervention, stabilization and transport of the sick and injured to the hospital in emergency situations. Also, recruitment of paramedics in the ambulance service might have prevented untimely death in this particular case.

## Introduction

Firearms are regarded as dangerous weapons in forensic practice. Firearm-related injuries are considered a major problem globally ^1^. Annual firearm injury rate was 8.8/100000 population in Canada of which 5.5/100000 were unintentional among children and youths aged less than 24 years ^2^. Possession of a firearm without a certified valid license is against Nepalese law. It is a cognizable offense to keep a firearm or ammunition. After a decade-long civil war in Nepal, amendments were made to the Arms and Ammunition Act 1962 (second amendment 2007), which included a clause that states: “Government of Nepal may, if it deems fit for the sake of public safety, give an order to withhold the arms or ammunition after having them seized anytime although anybody has a license to have in his/her possession the arms or ammunition”. This rule came into effect immediately and the public were instructed to submit their firearms to the nearest police station. To control the civil war, the law was mandated for the public. However, there are still people who possess firearms in their homes ^3^. Furthermore, hunting wild animals is cognizable offence in Nepal. Smuggling of hides and body parts of wildlife is a ludicrous business. Although the actual statistics are lacking in these regards, there have been cases of firearm injuries and fatalities as reported by Nepal police in their official webpage ^4^. Nepalese police have further reported arrests made for possession of hides and body parts of wildlife ^4^. Although illegal, the hunting of wild animals is quite common in rural Nepal, deer being the most highly prized trophy animal.

Nepal is a developing country in the south-east Asia region with a population of 29.1 million as per the census of 2021 ^5^. There are 28477 registered doctors in Nepal till December 31, 2020 ^6^. This country has 125 public hospitals, 196 primary health care centers (PHCC), and 3806 health posts (HP) ^7^. Health posts are a first contact point for health services especially in the rural areas, however these HPs lack a doctor and are run by paramedics. The present case highlights the medical issue in which the ambulance service in rural Nepal lacks a paramedic. No intervention in the form of life support and prompt resuscitation is a reason for more lives lost on the way to the hospital.

## Case report

### Circumstance

**Figure 1.**
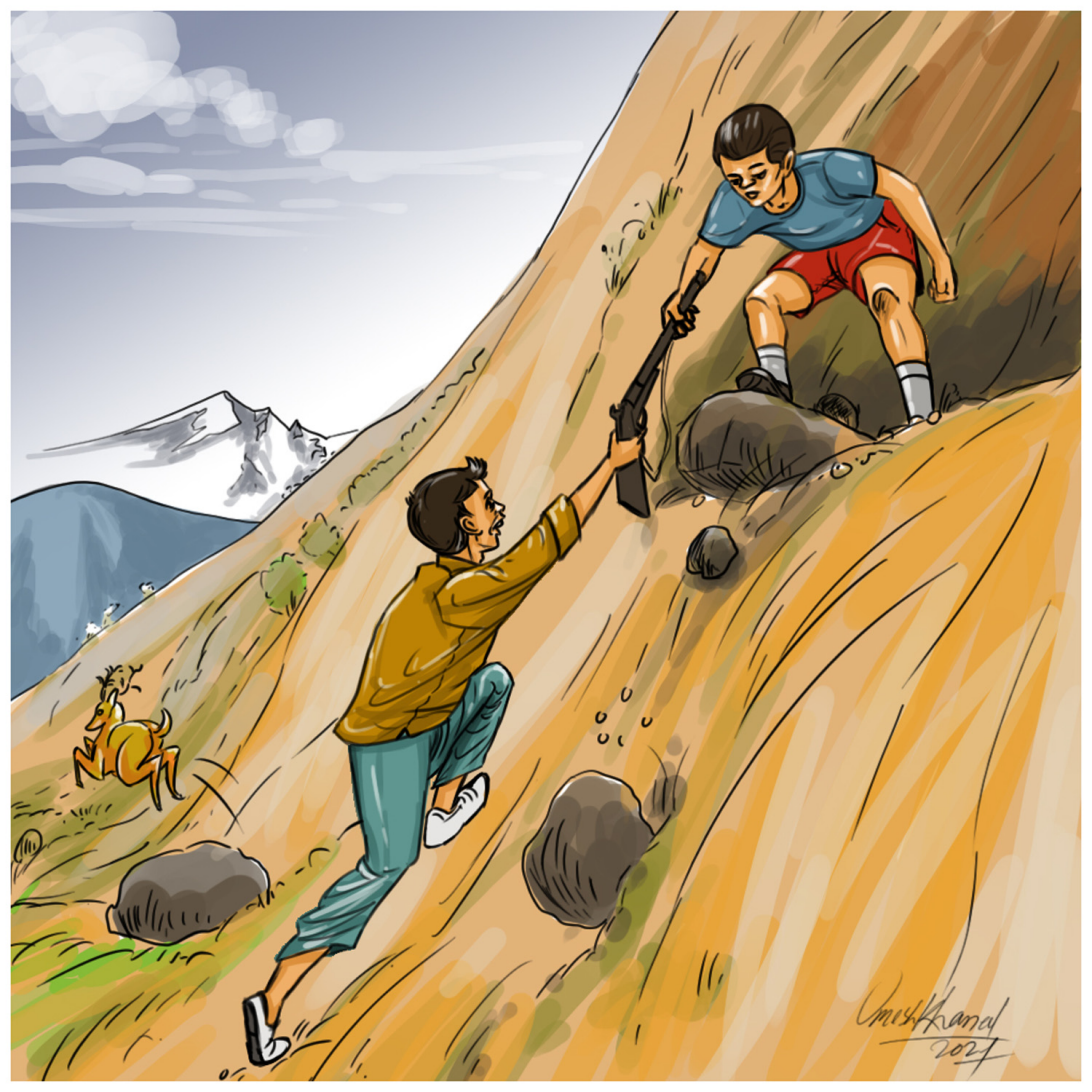
Graphical illustration of the scene of the incident.

During late January, a 13-year-old schoolboy from the Palpa district in Nepal planned hunting with four friends from the neighborhood. They decided to sneak out after their supper late in the evening. Due to the cold winter weather, people in remote mountainous village of the Palpa district usually sleep at around 8 pm. The boys gathered at around 9 pm and geared up for the hunt. The 13-year-old schoolboy led the group carrying a musket. After entering the dense woods and climbing uphill they spotted a deer with their torch. The leading boy who brought the firearm fired a shot at the deer with the pre-loaded gun. Although the deer was hit, it managed to run away. Knowing that the deer was wounded, the boys thought the deer would not run far. They reloaded the musket and followed the injured animal. They had to leave the usual trail and ran down rough terrain. At one point there was a big drop with large, jagged rocks lying at the bottom ( Figure 1). The boy carrying the gun handed it to the boy behind him and climbed down first. He was then passed the gun held at the muzzle end. After grasping the gun near the butt end with his right hand, he pulled the gun towards himself whilst trying to hold it near the mid-length with the left hand, however, there was a sudden slight thrust of the gun butt on a rock and the gun went off, shooting the boy.

The injured boy was rescued by the family members and the villagers within an hour of the incidence. He was then brought home. An ambulance was called, which arrived 45 minutes later to take him to the hospital, but before he could reach the hospital, he breathed his last breath the same night. In the present case, the patient was bleeding profusely through the wound on the chest. No intervention in the form of first-aid or basic life support was provided to the patient who died while being transported to the hospital.

**Figure 2.**
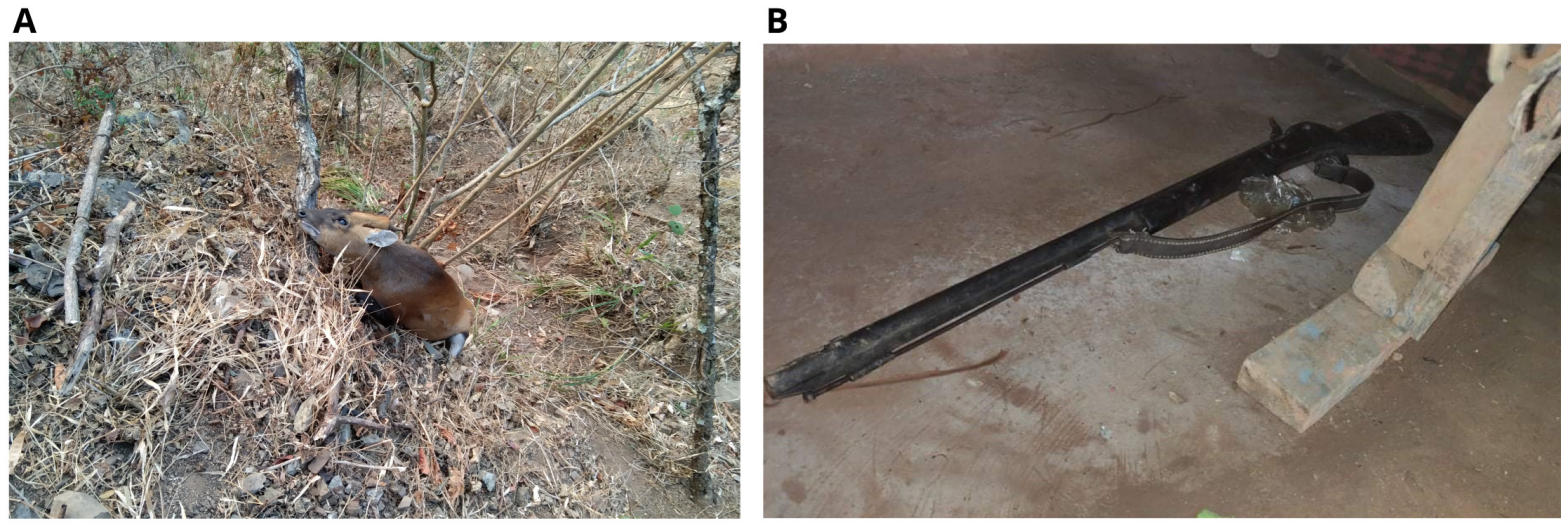
Dead barking deer ( **A**) and the gun used for hunting ( **B**).

For the validity of the statements made by the boys, police deployed a search team and found a dead male barking dear 400 m from the scene of the crime ( Figure 2).

### Autopsy

**Figure 3.**
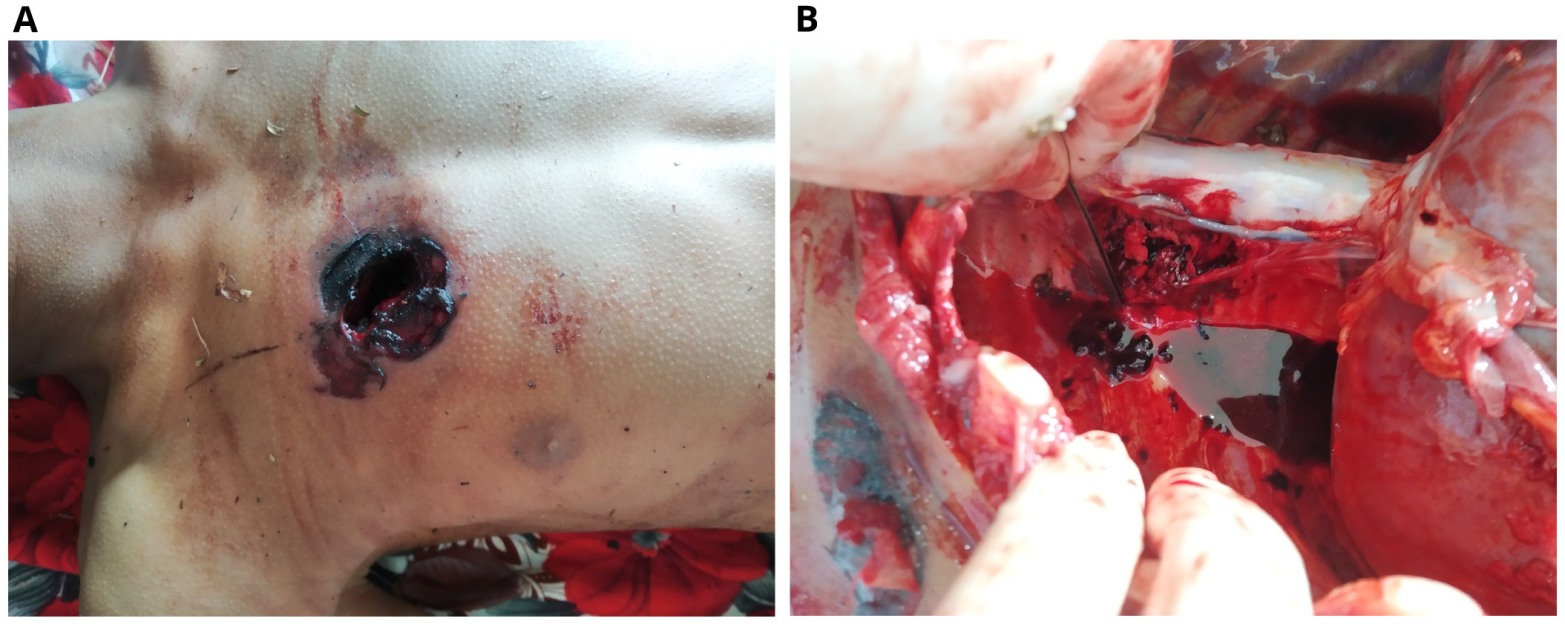
Entry wound in the front of the chest ( **A**). The location of the shot in the thoracic spine ( **B**).

The dead body of a 13-year-old boy was brought for autopsy. The body was cold to touch and stiff at all joints. Postmortem lividity couldn’t be seen on the back. There was an elliptical blackish laceration, suggestive of an entry wound of projectile, with abraded margin present over the right anterior chest having a maximum diameter of 3.5 cm ( Figure 3). This wound was located 35 cm from the vertex, 108 cm from the heel of the right foot, 5 cm from the mid-sternal point in the midline, 7 cm from the sternal notch, and 13 cm from the mid-axillary line. There was a tattooing around the wound over an area of 7 x 5 cm. Following skin incision an oval wound was present over the right third intercostal space with a maximum diameter of 3 cm, the margin of which was burnt and black in color. There was contusion of surrounding intercostal muscles present. The right lung was collapsed with a laceration on the upper and middle lobes. The left lung appeared normal. The shot was lodged in between the fourth and fifth thoracic vertebrae on the right. There was no exit wound.

## Discussion

Paramedics are the experts who provide emergency medical care and stabilize the patient before and during transportation to tertiary care hospital for further management. However, in rural Nepal paramedic service is not available. Whenever an ambulance is called, it the ambulance driver who comes to receive the patient and then transports them to hospital. Due to the lack of first aid, stabilization, maintaining vitals and resuscitation in timely manner more sick and injured die on the way to hospital ^8^. Prompt intervention using basic medical skills too can prolong survival and limit the complications. In high-income countries the role of paramedics has evolved to be competent to provide emergency medical services such as advance life support, acute presentations of mental illness and wide range of urgent-care presentations ^9^. In the present case hemorrhage and collapsed right lung causing respiratory compromise contributed to death. Fluid resuscitation is a gold-standard strategy in early management of traumatic injury which involves loss of body fluid. In cases of severe bleeding, blood transfusion should be done; in the absence or non-availability of blood, fluid replacement with crystalloids or colloids is an alternative ^10^. In the present case had the paramedic secured an IV line and gave fluid, provided oxygen and had accompanied the patient till he reached the tertiary care, the outcome might had been different.

As per the ‘Arms and Ammunition Act’ of the Nepalese legislature, it is prohibited to possess or carry any type of arms and ammunition without proper license or contrary to the terms and conditions specified in the license. If anyone is found guilty, the offender can be arrested without a warrant, the arms and ammunition seized, and can be punished with three to five years of imprisonment and a fine ranging from 60 to 100,000 Nepalese rupees ^11^.

Hunting of wildlife is prohibited in Nepal. If anyone wishes to hunt wildlife, an application is to be submitted to the concerned authority. All the details including the type of arms to be used, license number, licensing authority, and the number of hunters is to be mentioned. After evaluation of the application, the permit is issued with applicable fees. The type of animal that can be hunted, the number of animals that can be killed, and the method of hunting are specified in the permit ^12^. Nepal is also one of the popular destinations for trophy hunting for international tourists. There are several packages for trophy hunting in Nepal for foreign nationals, the trophy animals being blue sheep and Himalayan thar ^13^.

Most accidental gunshot injuries inflicted while hunting occur in the chest. The injuries are usually a result of a two-party accident and the victim is a relative or friend of the shooter ^14^. Gun handling by children is a well-known risk factor for such unintentional accidents ^15^. Young children residing in rural areas are the most affected group ^16^.

The most common weapon responsible for accidental injuries is a shotgun and the shots are of close range ^14^. Such shots are so dangerous that they cause extensive multisystem damage and produce long-term sequelae in survivors ^17^.

A study in the US showed that unintentional firearm injuries most commonly involve a combination of home guns and the young male population. The study also suggested a majority of injuries could be prevented by avoiding children’s access to guns ^18^. Another study suggests keeping guns locked and unloaded as a preventive measure ^19^. Guns are usually recovered from the house of the victim, relative, or friend ^20^. Those who acquire firearms are at increased risk of not only accidental but also suicidal deaths as a consequence of the impulsive act ^21^.

## Conclusions

In the present case, a dead male barking deer was recovered. Shooting on target and precise knowledge of handling the musket and muzzle loading technique point towards the fact that the hunting activities were common in the family and neighborhood. Although the deceased’s father said that he was not aware that his son had gone hunting, it is obvious that any of the four adolescent boys didn’t take permission from their families for the hunting trip. Furthermore, a gun is not a toy or a souvenir to be kept open and within the reach of the children. The gun was kept illegally without a gun license. The gun law in Nepal is stringent, however, this case highlights the fact that the law is defied, and firearms are kept in possession for trophy hunting in rural mountains. Illegal activity has negative consequences sooner or later, in this case, it was a life lost.

The victim in the present case succumbed to death before he reached to the hospital. Monitoring of the vitals and providing basic life support would have prolonged the life of the victim in the present case. The authors also would like to bring to attention of the stake holders and policy makers on the issue in which the ambulance service in rural Nepal lack a paramedic. No intervention in the form of life support and prompt resuscitation is a reason for more life lost on the way to the hospital.

## Data availability

All data underlying the results are available as part of the article and no additional source data are required.

## Consent

Written informed consent for publication of their clinical details and/or clinical images was obtained from the deceased boy’s father.
