# Supplementary material for: Case Report: Accidental firearm injury during trophy hunting and the role of paramedics in managing such cases at rural health posts in Nepal
Source: F1000Res. 2022 Mar 4;10:893. Originally published 2021 Sep 6. [Version 2] doi: 10.12688/f1000research.55659.2 (PMC8855013; doi:10.12688/f1000research.55659.2)
Supplement: Peer review history

# Peer review: Reviewer response for version 2

**DOI:** 10.5256/f1000research.121535.r126278

**Published:** 2022-03-07

**Authors:** Gilbert Koome

**Affiliations:** 1 Mount Kenya University, Nairobi, Kenya

Changes are incorporated.

Are enough details provided of any physical examination and diagnostic tests, treatment given and outcomes?

Partly

Is the case presented with sufficient detail to be useful for other practitioners?

Partly

Is sufficient discussion included of the importance of the findings and their relevance to future understanding of disease processes, diagnosis or treatment?

Partly

Is the background of the case’s history and progression described in sufficient detail?

Partly

Reviewer Expertise:

Public health and emergency care

I confirm that I have read this submission and believe that I have an appropriate level of expertise to confirm that it is of an acceptable scientific standard.

# Peer review: Reviewer response for version 1

**DOI:** 10.5256/f1000research.59253.r123821

**Published:** 2022-02-17

**Authors:** Alessandro Leite Cavalcanti

**Affiliations:** 1 Departament of Dentistry, School of Dentistry, State University of Paraíba, Campina Grande, Brazil

The manuscript is well written and presents a detailed case report of a hunting accident.

 1) I am suggesting that the authors replace keywords with terms contained in MESH/PubMed. I even offer some suggestions:

- Wounds, Gunshot
- Accidental Injuries 
- Wounds, Penetrating

2) Regarding the introduction, despite bringing relevant information, I would like to suggest to the authors that they include some epidemiological data on the prevalence of firearm accidents. It can be in Nepal or in another country.

Are enough details provided of any physical examination and diagnostic tests, treatment given and outcomes?

Yes

Is the case presented with sufficient detail to be useful for other practitioners?

Yes

Is sufficient discussion included of the importance of the findings and their relevance to future understanding of disease processes, diagnosis or treatment?

Yes

Is the background of the case’s history and progression described in sufficient detail?

Yes

Reviewer Expertise:

Epidemiology and Public Health

I confirm that I have read this submission and believe that I have an appropriate level of expertise to confirm that it is of an acceptable scientific standard, however I have significant reservations, as outlined above.

# Response

**Published:** 2022-02-19

**Authors:** Alok Atreya

**Affiliations:** Lumbini Medical College, Nepal

*We wish to thank you all for your constructive comments in this review. Your comments provided valuable insights to refine its contents.*

 The manuscript is well written and presents a detailed case report of a hunting accident.

 1) I am suggesting that the authors replace keywords with terms contained in MESH/PubMed. I even offer some suggestions:

 ○ Wounds, Gunshot

 ○ Accidental Injuries

 ○ Wounds, Penetrating

*Response: The keywords are replaced as suggested.*

 2) Regarding the introduction, despite bringing relevant information, I would like to suggest to the authors that they include some epidemiological data on the prevalence of firearm accidents. It can be in Nepal or in another country.

*Response: As the prevalence of firearm accidents from Nepal is lacking we did include the prevalence of such accidents from Canada.*

# Peer review: Reviewer response for version 1

**DOI:** 10.5256/f1000research.59253.r121895

**Published:** 2022-02-09

**Authors:** Gilbert Koome

**Affiliations:** 1 Mount Kenya University, Nairobi, Kenya

While the case doesn’t present new knowledge in the field, it offers valuable insight on accidental fire-arm fatality in Nepal while underscoring possible driving factors for the potentially avoidable fatality. While the article makes a strong case for strengthening access to EMS at the prehospital care, the following aspects can be added or improved:

**Title –** The authors have substantially incorporated information around access to paramedic or EMS as gap and driver for the avoidable fatality from firearm accident. These aspects can be captured in the title of the article.

**Background - **The information is relevant and concise. However, it fails to provide a good picture of the EMS in Nepal, especially at the prehospital level or out of hospital settings in which this case study happened. This literature can be captured to provide a logical link to the discussions and conclusion that ensues around this issue.

**Findings and Discussions - **It would have been more valuable to add information on what led to the fatality in this case to help build the case for EMS or paramedics. In other words, based on autopsy findings, what led to death such as Exsanguination or combination of various factors? What role could EMS or paramedics play in avoiding this case fatality in respect to their professional roles?

**Conclusion:** Review and align with the key findings especially on the aspect of EMS which has been alluded to. The conclusion also fails to make a logical conclusion on the accidental firearm fatality and possible reasons, instead, it provides additional information around the case (both contextual information such as illegality of owning a firearm without license, defiance to law, parental law in safeguarding children safety etc).

Are enough details provided of any physical examination and diagnostic tests, treatment given and outcomes?

Partly

Is the case presented with sufficient detail to be useful for other practitioners?

Partly

Is sufficient discussion included of the importance of the findings and their relevance to future understanding of disease processes, diagnosis or treatment?

Partly

Is the background of the case’s history and progression described in sufficient detail?

Partly

Reviewer Expertise:

Public health and emergency care

I confirm that I have read this submission and believe that I have an appropriate level of expertise to confirm that it is of an acceptable scientific standard, however I have significant reservations, as outlined above.

# Response

**Published:** 2022-02-19

**Authors:** Alok Atreya

**Affiliations:** Lumbini Medical College, Nepal

We wish to thank you all for your constructive comments in this review. Your comments provided valuable insights to refine its contents.

 Comments: While the case doesn’t present new knowledge in the field, it offers valuable insight on accidental fire-arm fatality in Nepal while underscoring possible driving factors for the potentially avoidable fatality. While the article makes a strong case for strengthening access to EMS at the prehospital care, the following aspects can be added or improved.

**Title – **The authors have substantially incorporated information around access to paramedic or

 EMS as gap and driver for the avoidable fatality from firearm accident. These aspects can be

 captured in the title of the article.

*Response: The title has been changed.*

**Background - **The information is relevant and concise. However, it fails to provide a good picture

 of the EMS in Nepal, especially at the prehospital level or out of hospital settings in which this case

 study happened. This literature can be captured to provide a logical link to the discussions and

 conclusion that ensues around this issue.

*Response: The information is incorporated.*

**Findings and Discussions - **It would have been more valuable to add information on what led to

 the fatality in this case to help build the case for EMS or paramedics. In other words, based on

 autopsy findings, what led to death such as Exsanguination or combination of various factors?

 What role could EMS or paramedics play in avoiding this case fatality in respect to their

 professional roles?

*Response: We have added the probable cause of death and have discussed the role of paramedics in managing such cases.*

**Conclusion: **Review and align with the key findings especially on the aspect of EMS which has been alluded to. The conclusion also fails to make a logical conclusion on the accidental firearm fatality and possible reasons, instead, it provides additional information around the case (both contextual information such as illegality of owning a firearm without license, defiance to law, parental law in safeguarding children safety etc).

*Response: We have now incorporated the findings on the aspect of EMS.*

# Peer review: Reviewer response for version 1

**DOI:** 10.5256/f1000research.59253.r94127

**Published:** 2021-09-20

**Authors:** Vikash P. Meshram

**Affiliations:** 1 Department of Forensic Medicine and Toxicology, All India Institute of Medical Sciences, Jodhpur, Rajasthan, India

This article describes the circumstances of accidental firearm fatality involving a schoolboy with an illegal firearm, despite stringent Arms and Ammunition laws in Nepal. The authors have emphasized the need for the mandatory presence of medical personnel in the ambulance and the significance of emergency care in a golden hour. Overall, the article is well written. Graphical representation nicely depicts the circumstances of the incident. Information about the examination of clothes, cause of death and a possible range of the firearm injury are to be added in the manuscript. ‘Emergency care’ can be added in the keywords. Please clarify about the presence of tattooing in the injury. I could appreciate sparsely visible tattooing in the left upper part of the injury. The article can be approved for indexing after minor revision.

Are enough details provided of any physical examination and diagnostic tests, treatment given and outcomes?

Yes

Is the case presented with sufficient detail to be useful for other practitioners?

Yes

Is sufficient discussion included of the importance of the findings and their relevance to future understanding of disease processes, diagnosis or treatment?

Yes

Is the background of the case’s history and progression described in sufficient detail?

Yes

Reviewer Expertise:

Forensic Pathology, Sexual Jurisprudence, Forensic Anthropology, Clinical Forensic Medicine, Toxicology

I confirm that I have read this submission and believe that I have an appropriate level of expertise to confirm that it is of an acceptable scientific standard.

# Response

**Published:** 2022-02-19

**Authors:** Alok Atreya

**Affiliations:** Lumbini Medical College, Nepal

We wish to thank you all for your constructive comments in this review. Your comments provided valuable insights to refine its contents.
